# Tracheal Spindle Cell Lipoma: A Case Report

**DOI:** 10.70352/scrj.cr.26-0305

**Published:** 2026-08-25

**Authors:** Kazuya Kishimoto, Ryu Nakajima, Kyukwang Chung, Satoshi Okada, Hiroyuki Kochi, Takuma Tsukioka

**Affiliations:** Department of General Thoracic Surgery, Osaka City General Hospital, Osaka, Osaka, Japan

**Keywords:** spindle cell lipoma, tracheal neoplasms, bronchoscopic resection, bronchoscopy, airway obstruction

## Abstract

**INTRODUCTION:**

Spindle cell lipoma (SCL) is a rare benign adipocytic tumor that typically arises in the subcutaneous tissue of the posterior neck, shoulder, and upper back. Occurrence in the airway is exceedingly rare, with only a few cases reported in the literature.

**CASE PRESENTATION:**

A 39-year-old man presented with an 8-month history of progressive cough and dyspnea on exertion. CT revealed a tumor occupying the tracheal lumen. Rigid bronchoscopy demonstrated a well-defined, smooth-surfaced, mobile tumor arising from the right lateral tracheal wall. The tumor was removed by rigid bronchoscopy, and no visible residual lesion was observed bronchoscopically after resection. Histopathological examination showed bland spindle cells admixed with mature adipocytes and ropey collagen bundles. Immunohistochemical analysis demonstrated diffuse CD34 positivity, supporting the diagnosis of SCL.

**CONCLUSIONS:**

SCL can rarely arise in the airway and may cause clinically significant airway obstruction despite its benign histology. Preoperative diagnosis by bronchoscopic biopsy may be difficult, as these lesions are typically well-circumscribed and smooth-surfaced. Therefore, this entity should be considered in the differential diagnosis of benign-appearing airway tumors, and timely intervention may be required to prevent potential airway compromise.

## Abbreviation

SCL
: spindle cell lipoma

## INTRODUCTION

SCL is a rare benign adipocytic tumor that typically arises in the subcutaneous tissue of the posterior neck, shoulder, and upper back and predominantly affects middle-aged to older men.^1,2)^ In contrast, occurrence in the airway is exceedingly rare, with only a few cases reported in the literature.^3–5)^

Airway tumors, even when histologically benign, can cause clinically significant symptoms due to luminal obstruction.

Here, we report a rare case of SCL arising in the trachea and discuss its clinical characteristics with a review of the literature.

## CASE PRESENTATION

A 39-year-old man presented with an 8-month history of progressive cough and dyspnea on exertion. Chest CT revealed a tumor occupying the tracheal lumen. He was referred to our institution for further management.

**Fig. 1:**
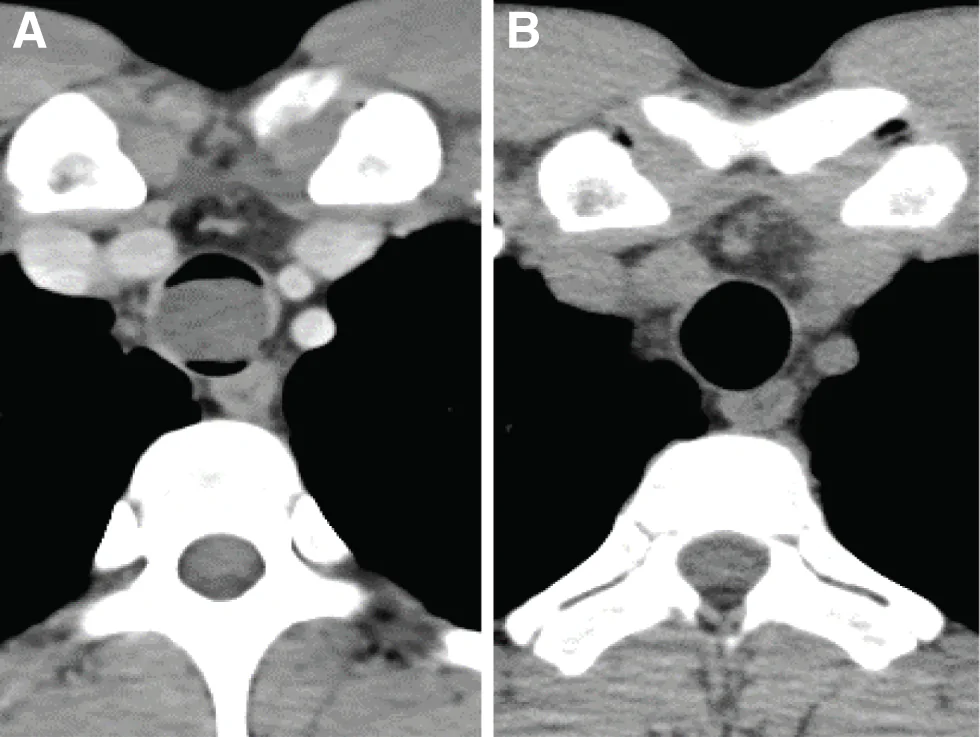
CT findings. (**A**) Preoperative chest CT. A 2.2 × 2.0-cm intraluminal tumor is observed in the trachea approximately 6.0 cm above the carina. (**B**) Postoperative chest CT. No definite residual tracheal lesion is observed after bronchoscopic resection.

**Fig. 2:**
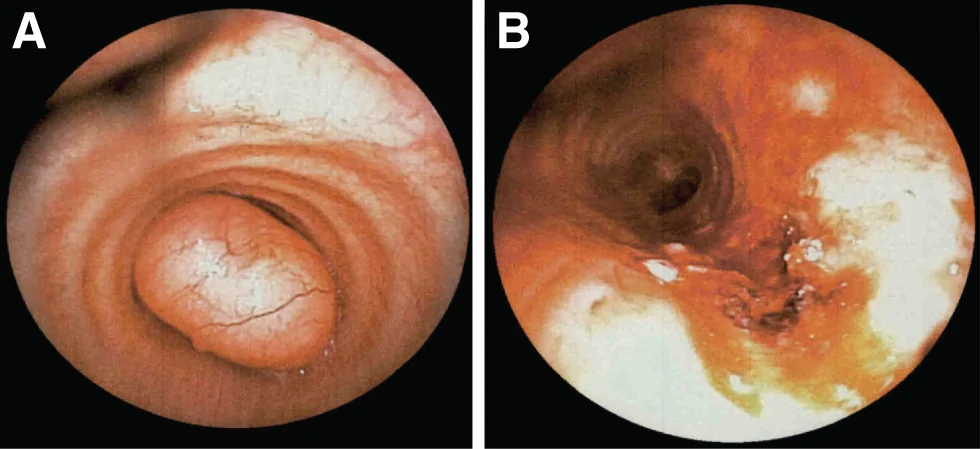
Bronchoscopic findings before (**A**) and immediately after (**B**) bronchoscopic resection. (**A**) A smooth-surfaced pedunculated tumor nearly obstructing the tracheal lumen is observed. (**B**) No apparent residual intraluminal tumor is observed after bronchoscopic resection.

On admission, vital signs were within normal limits, and inspiratory stridor was audible over the neck. Chest CT revealed a 2.2 × 2.0-cm tumor in the trachea approximately 6.0 cm above the carina (**Fig. 1A**). The tumor was well-defined, smooth-surfaced, and mobile, with attachment to the right lateral tracheal wall over a limited area (**Fig. 2A**). Rigid bronchoscopy was performed under general anesthesia for both diagnosis and treatment. The tumor was a large pedunculated lesion arising from the right lateral tracheal wall. Because stable grasping of the smooth-surfaced tumor was difficult, the lesion was detached from the tracheal wall using a debulking technique. The tumor was then retrieved with forceps; however, because of its large size, en bloc retrieval was difficult, and the specimen was removed piecemeal. As a result, the entire tumor was not submitted as a single oriented specimen suitable for histological margin assessment, and a tumor-free resection margin could not be confirmed pathologically. Bleeding was minimal and was controlled endoscopically using electrocautery coagulation. Immediate post-resection bronchoscopy demonstrated no visible residual lesion (**Fig. 2B**). Postoperative CT also demonstrated no definite residual tracheal lesion (**Fig. 1B**). Postoperative symptoms resolved completely, and the cervical stridor disappeared. The patient was discharged on POD 2 in good condition. No recurrence has been observed during 9 months of follow-up.

**Fig. 3:**
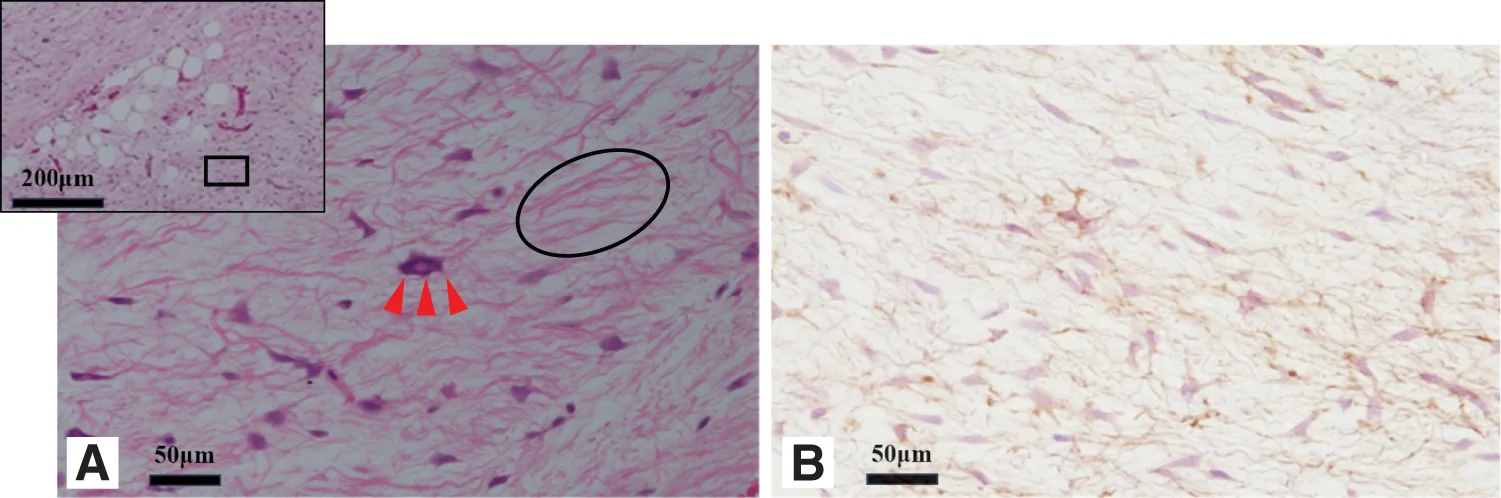
Histological and immunohistochemical findings. (**A**) Hematoxylin and eosin staining. The inset shows a low-magnification view, demonstrating clusters of mature adipocytes. The main panel shows a higher-magnification view of the boxed area, revealing bland spindle cells (red arrowheads) within a collagenous stroma. Thick, eosinophilic “ropey” collagen bundles (black circle) are also evident. (**B**) CD34 immunohistochemistry. Bland spindle cells show diffuse cytoplasmic positivity for CD34.

Histopathological examination revealed bland spindle cells admixed with mature adipocytes within a collagenous stroma, including thick eosinophilic “ropey” collagen bundles (**Fig. 3A**). These features are characteristic of SCL. Immunohistochemical analysis demonstrated diffuse CD34 positivity in the spindle cells (**Fig. 3B**), focal S-100 positivity, and negativity for MDM2, further supporting the diagnosis of SCL.

## DISCUSSION

SCL is a rare benign adipocytic tumor first described by Enzinger and Harvey in 1975^1)^ and accounts for approximately 1%–2% of all adipocytic tumors.^2)^ SCL predominantly affects men between the ages of 40 and 70 and typically arises in the subcutaneous tissue of the posterior neck, shoulder, and upper back,^2)^ whereas occurrence in the airway is exceedingly rare.

Only a few cases of SCL arising in the airway (airway SCL) have been reported in the literature, and the present case adds to this limited body of evidence.^3–5)^ A review of the 4 reported cases, including the present case, reveals several shared clinical characteristics of airway SCL (**Table 1**). All patients were male, ranging in age from their 30s to their 80s, consistent with the known male predominance of SCL in conventional subcutaneous sites.^2)^ The most common presenting symptoms were cough and dyspnea on exertion. The tumors were located in the trachea or central bronchi and measured approximately 1.0–2.2 cm in diameter. Despite their benign histology, these lesions were sufficiently large to result in clinically significant airway obstruction.

**Table 1:** Summary of reported cases of airway spindle cell lipoma

Author (Year)	Age (Years)	Sex	Location	Size (cm)	Symptoms	Treatment	Prognosis
Sakr et al.^5)^ (2008)	70	Male	Trachea	1.8	Cough	Rigid bronchoscopic resection using high-frequency snare	NA
Rodriguez et al.^3)^ (2020)	61	Male	Right middle lobe bronchus	1.0	Cough, DOE	Flexible bronchoscopic resection using high-frequency snare	NA
Krishnan et al.^4)^ (2022)	72	Male	Left main bronchus	NA	Cough, DOE	Flexible bronchoscopic resection using cryotherapy and argon plasma coagulation	NA
Present case (2025)	39	Male	Trachea	2.2	Cough, DOE	Rigid bronchoscopic resection using electrocautery with a coagulation technique	Alive without recurrence at 9 months

DOE, dyspnea on exertion; NA, not available

Prognostic data have not been reported in previously described cases of airway SCL. In the present case, no recurrence has been observed during the 9-month follow-up period. Given the excellent prognosis of SCL following complete excision,^1,2)^ airway SCL is expected to follow a similar clinical course. Nevertheless, further case accumulation and long-term follow-up are necessary to better characterize its clinical course. However, the optimal duration and modality of follow-up for airway SCL remain unclear because of the limited number of reported cases and the lack of long-term outcome data. At least several years of periodic follow-up with imaging and/or bronchoscopy may be reasonable, particularly in cases treated bronchoscopically without definitive histological margin assessment. In the present case, pathological margin assessment was not possible because the tumor was removed piecemeal.

In 2 previously reported cases, flexible bronchoscopy with forceps biopsy did not yield a definitive diagnosis prior to resection.^3,4)^ SCL is typically described as a circumscribed mass composed of mature adipocytes and bland spindle cells.^1)^ In a previously reported airway case, the lesion appeared as an exophytic, sessile polypoid mass on bronchoscopy,^3)^ and in the present case, bronchoscopy demonstrated a well-defined, smooth-surfaced intraluminal tumor. Furthermore, benign endobronchial lipomas have been reported to be covered by intact respiratory epithelium^6)^; therefore, if airway SCL shares similar characteristics as a variant of lipoma, superficial biopsy may fail to capture representative spindle cell components, resulting in a limited diagnostic yield. Therefore, when a well-defined, smooth-surfaced intraluminal lesion raises suspicion for airway SCL, establishing a definitive diagnosis by bronchoscopic biopsy may be challenging. Taken together, these findings suggest that tracheal SCL should be considered in the differential diagnosis of benign-appearing intraluminal airway tumors.

Symptom severity does not necessarily reflect the risk of airway obstruction. Tumors with high mobility or a pedunculated morphology may intermittently obstruct airflow. A previous report of a benign endobronchial tumor demonstrated that a mobile, pedunculated lesion can shift with respiration, resulting in obstruction at different airway sites during inspiration and expiration.^7)^ Therefore, careful assessment of tumor morphology and mobility is essential. In the present case, despite relatively mild symptoms, the tumor’s mobility suggested a potential risk of airway obstruction, supporting the decision to perform early intervention to prevent unexpected airway compromise.

## CONCLUSIONS

SCL can rarely arise in the airway and may cause clinically significant airway obstruction despite its benign histology. Preoperative diagnosis by bronchoscopic biopsy may be difficult, as these lesions are typically well-circumscribed and smooth-surfaced. Therefore, airway SCL should be considered in the differential diagnosis of benign-appearing airway tumors, and timely intervention may be required to prevent potential airway compromise.

## DECLARATIONS

### Availability of data and materials

The datasets supporting the conclusions of this article are included within the article.
